# Characterization of Biocompatible Hydrogel Lenses Using Methacrylic Acid with Neodymium Oxide Nanoparticles

**DOI:** 10.3390/polym13101575

**Published:** 2021-05-14

**Authors:** Min-Jae Lee, Seon-Young Park, A-Young Sung

**Affiliations:** 1 Department of Optometry, Jeju Tourism University, Jeju 63063, Korea; tomssamac@naver.com; 2 Department of Optometry & Vision Science, Daegu Catholic University, Gyeongsan 38430, Korea; ssun_419@naver.com

**Keywords:** neodymium oxide nanoparticles, methacrylic acid, hydrogel contact lens, tensile strength, wettability

## Abstract

We prepared hydrogel contact lenses containing nanoparticles of neodymium oxide and methacrylic acid (MA) to investigate their effect on the physical and chemical properties of the lens. Neodymium oxide nanoparticles improved the tensile strength without affecting wettability. The tensile strength, wettability, and light transmittance were all increased when MA was added in a specific ratio. To confirm the safety of the newly used nanoparticles, test on absorbance, eluate, and pH change were conducted and it was found that the safety level was satisfactory. In conclusion, it was confirmed that durable contact lenses can be manufactured with neodymium oxide nanoparticles, and most of the basic elements of the lens such as transparency, strength, and wettability could be improved using MA, which is a hydrophilic material. It is believed that the study will be helpful as part of basic research to use new materials.

## 1. Introduction

Various types of hydrogel lens products such as disposable contact lenses, disposable astigmatism lenses, silicone hydrogel lenses, multifocal contact lenses, and reverse geometry lenses are available in the market today. The materials used for making these lens products are also changing [1]. Disposable and cosmetic contact lenses are particularly growing in popularity since they are very convenient to manage [2]. However, since hydrogel lenses come into direct contact with the surface of the cornea of the eyeball, only hydrogel lenses that ensure proper maintenance of the physiological function of the cornea should be worn [3]. Thus, when using disposable hydrogel lenses, uncomfortable feeling such as pain, sensation of a foreign body, dryness, glare, and redness should be minimized [4]. To this end, when selecting disposable hydrogel lenses, the type of lens and its oxygen permeability, contact angle, wettability, friction coefficient, manufacturing method, and corrective refractive power should be considered. Despite the many technologies now being used for developing the materials and design of hydrogel lenses, they still cause problems such as dryness and discomfort. Water content can be cited as one of the various factors related to this problem. Although hydrogel lenses with low water content are easier to handle than those with high water content, their oxygen transmissibility is relatively low, causing corneal edema that affects the scattering of light and the refractive power of the cornea and gives it a tendency to form poorer-quality images [5,6]. To address this problem, a hydrogel lens made of silicone mixed with an existing hydrogel material has been developed and is being distributed, but this new lens presents a possibility that the wettability of the lens will deteriorate due to the hydrophobicity of silicone [7]. In addition, it is known that dehydration of soft hydrogel lenses reduces visual acuity and fit condition [8]. This is because the dehydration of the contact lens subtly changes the shape of the lens [9], and the deformed hydrogel lens disturbs the tear layer [10] that ultimately causes optical scattering and aberration [11,12]. Therefore, in order to secure eye health and functionality, new hydrogel lens materials are being continuously developed and reported. The hydrogel may contain a large volume of water and the biocompatibility is excellent. This advantage has been used to mainly use it as a biomechanical material for treatment of wounds. The development of therapeutic material, which uses antimicrobial substances such as thaizole to form hydrogel materials, is still underway [13,14,15,16,17]. In this case, unlike the contact lens, it is not necessary to ensure the transparency of the material [18]. However, in the case of contact lenses, the basic purpose of visual acuity should be achieved. The hydrogel widely used as a contact lens material is recently been studied in drug emission medical lenses and several research studies have been conducted. Various antimicrobial and antiviral agents have been developed to satisfy the purpose of visual acuity while maintaining conformity with hydrogels, the base material of the lens [19,20,21,22]. In addition to changes in the material, efforts are in progress to minimize the impact of protein deposition that can occur in a continuous lens by changing the lens design [23,24]. Various efforts are being made to develop this new functionality [25]. In particular, in the case of nanoparticles, the physical properties are largely different depending on the type and size of the particles and sizes. There are several studies applying nanoparticles to contact lens materials, but it has been difficult to synthesize these particles and materials so as to secure compliance between materials [26,27,28,29]. However, the new materials must be compatible with the basic material of existing ophthalmic lenses: the hydrogel. This study applied a new nanoparticle to the hydrogel and investigated its physical and chemical properties upon controlling the additive to optimize the properties of the lens.

## 2. Materials and Methods

### 2.1. Reagents and Materials

For the reagents used in this study, including 2-hydroxyethyl methacrylate (HEMA) and azobisisobutyronitrile (AIBN), the products of Junsei were used, and methacrylic acid (MA), ethylene glycol dimethacrylate (EGDMA), Poly (ethylene glycol) methyl ether methacrylate (PEGMA), and Neodymium (III) oxide nanoparticles were purchased from Sigma-Aldrich (Saint Louis, MO, USA) and used without purification.

### 2.2. Polymerization

The sample formulations used in the polymerization are shown in Table 1. AIBN (0.2 wt%) was used as an initiator, and EGDMA (0.5 wt%) and PEGMA (10 wt%) were used as crosslinking agents. Lens samples were fabricated by varying the weight percent (%) of the MA monomer to 1~10%. The experimental group without using additives was named Ref. For the improvement of the physical properties of the lens, the combinations of PEGMA added at 10% were named P. In addition, the experimental groups, in which the Neodymium (III) oxide nanoparticles were added at 0.1% was named PN. The combinations with MA added to the PN sample at a ratio of 1 to 10% were named PNM1, PNM3, PNM5, and PNM10, respectively. After each sample was quantified in a vial, each mixed solution was stirred with a Vortex GENIE 2 stirrer (Scientific Industries, Bohemia, NY, USA) for 30 min at room temperature and then ultrasonicated for 30 min. The lens was fabricated using the cast-mold method and copolymerized for 90 min at a constant temperature of 100 °C. After the fabricated lens cooled down to room temperature, the copolymerized lens was separated from the mold. The fabricated hydrogel samples were hydrated in 0.9% physiological saline for 24 h, after which the physical properties of the lenses were measured and compared.

### 2.3. Analysis

The measurement standard for the refractive index was based on ISO 18369-4:2006. The refractive index of a lens sample hydrated in a physiological saline solution for 24 h was measured using an ABBE Refractometer (ATAGO DR-A1, Tokyo, Japan). The water content was determined using the gravimetric method provided by ISO 18369-4:2006. First, weight of the dried lens was measured before hydration, and then the lens was hydrated in water for 24 h, after which only the moisture on the surface of the lens was removed. Next, the mass of the hydrated lens was measured. This procedure was repeated five times for each lens sample, and the results were averaged. Agilent (Cary 60 UV-vis, Santa Clara, CA, USA) was used to measure the spectral transmittance of the UV-B (280–315 nm), UV-A (315–380 nm), and visible light regions (380–780 nm) five times each, and the results were averaged. Wettability was evaluated by measuring the contact angle (Contact Angle Instruments, (Kruss GMBH, DSA30, Hamburg, Germany)) using the Sessile drop method. The condition of the surface and roughness of the lens were measured using a scanning electron microscope (FESEM; JSM-7500F + EDS, JEOL, Tokyo, Japan) and an atomic force microscope (XE-100, Park Systems, Suwon, Korea). The absorbance, pH change, and presence or absence of a potassium permanganate-reducing substance was measured to check extractables. The pH change was deemed to have had no effect when the difference was less than 1.5 compared to the control group. Furthermore, in the case of the potassium permanganate-reducing substance, when the difference was less than 2 mL compared to the control group, it was deemed that there was no extractable.

## 3. Results and Discussion

### 3.1. Physical Properties

#### 3.1.1. Refractive Index and Water Content

**Figure 1:**
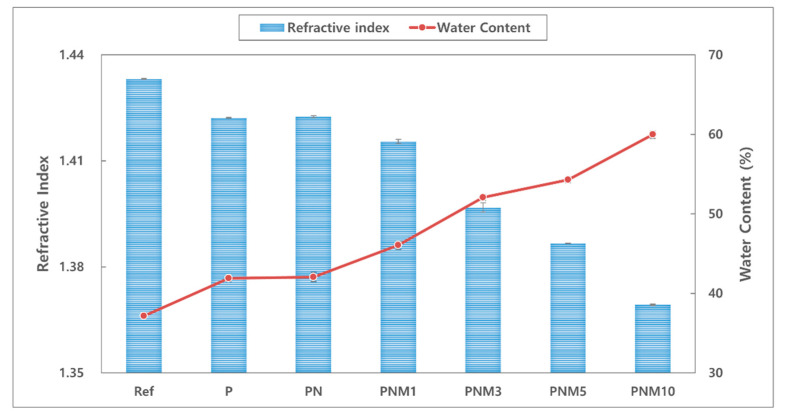
Change of refractive index and water content of samples.

The refractive index has an inverse relationship with the water content and the water content is lowered with decrease in the expansion rate [30]. In addition, water content is an important factor that has a great influence on the fitting comfort of contact lenses [31,32]. The refractive index of the fabricated hydrogel lens decreased from 1.433 to 1.369 when neodymium oxide nanoparticles and MA were added to the mixture. Furthermore, water content of the lens increased from 37.17% to 59.98%. These results are the same as those of previous studies, in which the refractive index was inversely proportional to the change in the water content due to the characteristics of the material [33]. Thus, the decrease in the swelling ratio is attributed to the increased density in the crosslink with a high substitution rate [34]. Therefore, neodymium oxide nanoparticles and MA are considered suitable to be used as wetting agents. The relationships between the refractive index and the water content of all the combinations are shown in Figure 1.

#### 3.1.2. Optical Transmittance

**Figure 2:**
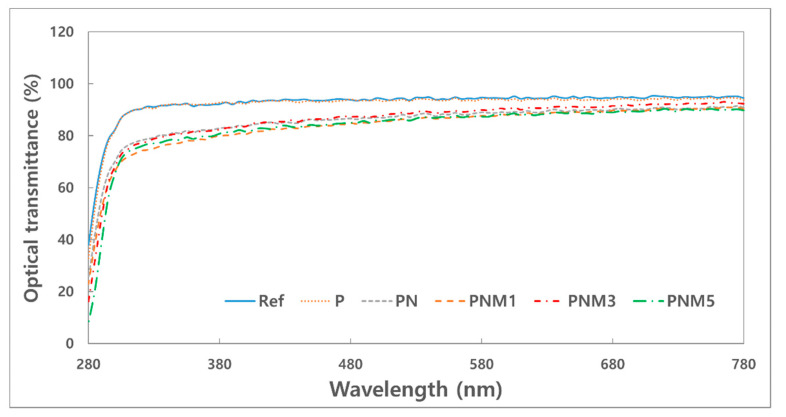
Optical transmittance of samples.

Figure 2 shows the spectral transmittance of the fabricated lens. The neodymium oxide nanoparticles reduced the transmittance of the UV-B, UV-A, and visible light. It was confirmed that the UV-B region was partially blocked by the addition of MA. Specifically, the addition of 3% MA improved the transmittance of visible light to 90.08%. As for spectral transmittance, ANSI Z80.20:2004 recommends an 88% or higher transmittance for a hydrogel contact lens. This standard for spectral transmittance can be satisfied with the addition of MA at an appropriate ratio.

#### 3.1.3. Tensile Strength

**Figure 3:**
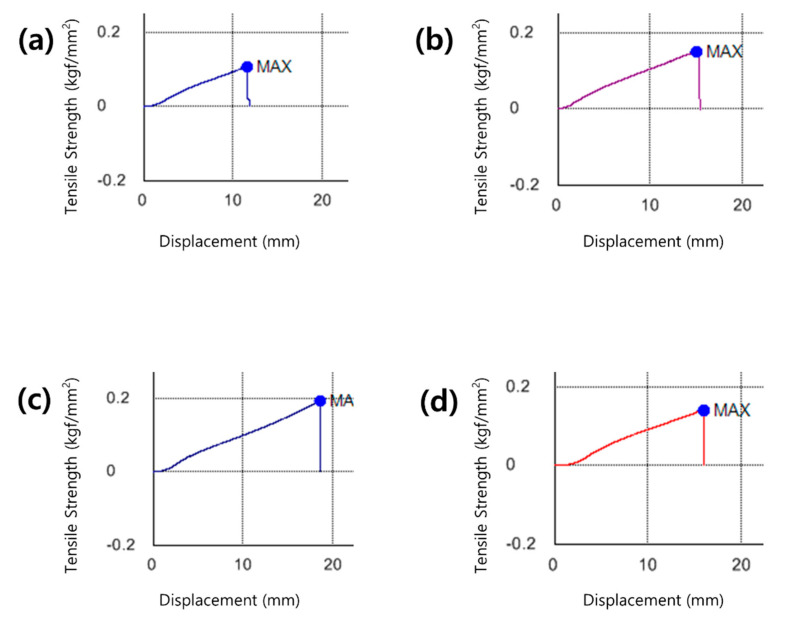
Tensile strength image of (**a**) P sample, (**b**) PN sample, (**c**) PNM3 sample and (**d**) PNM5 sample.

Lenses with high water content require caution to avoid damage such as tear of the lens during handling owing to low tensile strength [35]. There is a need for a contact lens that has excellent durability while maintaining the water content. The tensile strength increased by about 77.5% with the addition of neodymium oxide nanoparticles, increased again by 42.3% with the addition of 3% MA, and decreased with the addition of MA at a higher concentration. Regardless of the refractive index or water content of the lens, its tensile strength increased, so a further thermal analysis is deemed necessary. The tensile strengths of the samples are shown in the graph in Figure 3.

#### 3.1.4. Test for Absorbance and Extractables

**Figure 4:**
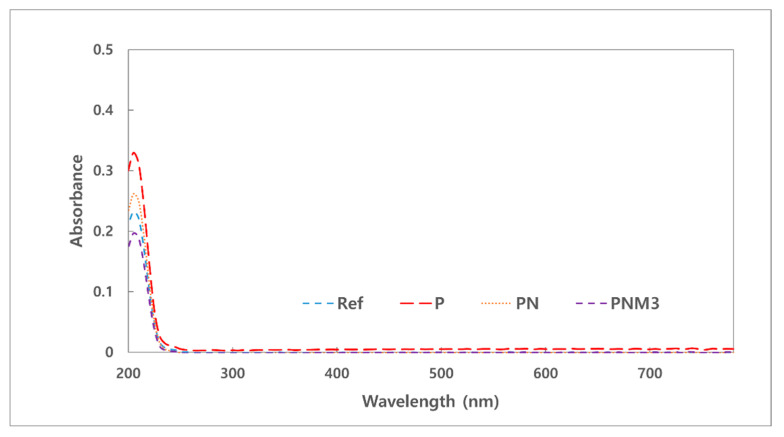
Absorbance of samples.

Medical devices need to be tested for extractables since they may harm the human body owing to the polymer additives that are eluted into the human body during contact [36]. Specifically, since polymers contain monomers, catalysts, polymerization initiators, and antioxidants in the fabrication process, they are highly likely to contain more ultraviolet-absorbing organic compounds [37]. There are several methods for evaluating the safety of medical devices, and the absorbance, extractable test, and pH changes were measured and evaluated according to the criteria [38]. The absorbance of the hydrated solution was 0.23 for Ref, 0.33 for P, 0.26 for PN, and 0.20 for PNM3. All the results showed the same trend as that in the extractable test, and the most stable value was shown in the PNM3 sample. Therefore, if MA is used as an additive, a 3% added amount can be considered stable. The measured absorbance values of the samples are presented in Figure 4. The results of the tests of the pH and potassium permanganate-reducing substance are shown in Table 2. The difference between the pH values of the fabricated lenses was less than 1.5 and had no effect. The difference between the amount of the potassium permanganate-reducing substance in the PNM3 sample and that in the control group was 2 mL or less, and the added nanoparticles and MA showed excellent compatibility with the other monomers used in the combination.

### 3.2. Surface Property

#### 3.2.1. Wettability

**Figure 5:**
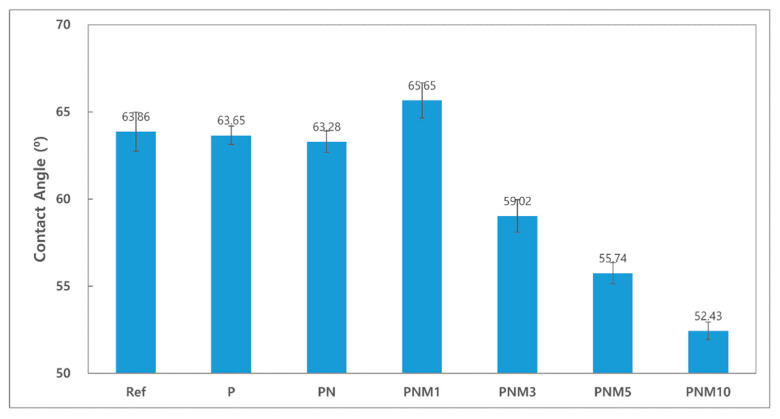
Contact angle of samples.

Contact lenses with good wettability tend to reduce dehydration and cause fewer tears [39]. The contact angles of the fabricated hydrogel lenses decreased from 63.86° to 52.43° based on the additives in all the groups. The wettability of the hydrogel lenses also tended to increase due to the hydrophilicity of its water content [40]. Since the contact angle of a general polyHEMA lens is known to be around 80°, the fabricated lenses were evaluated as highly wettable [41]. It is judged that the water content and wettability increased due to hydrogen bonding by hydroxyl groups of MA monomer [42]. The contact angles of the combinations are presented in Figure 5.

#### 3.2.2. SEM and AFM Analyses

**Figure 6:**
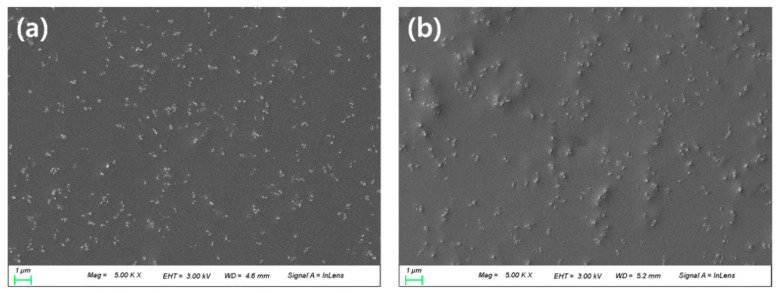
SEM analysis of (**a**) PN sample and (**b**) PNM3 sample.

**Figure 7:**
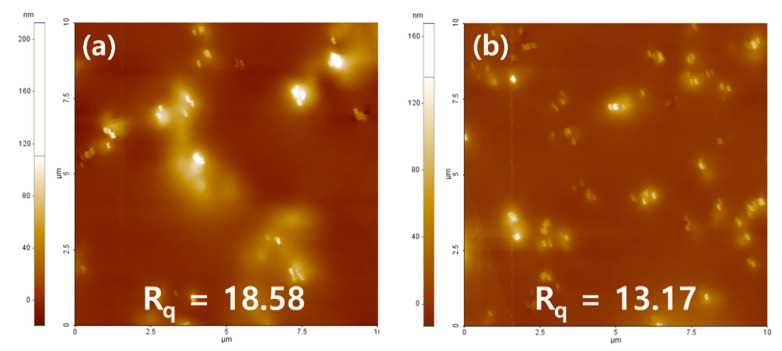
AFM analysis of (**a**) PN sample and (**b**) PNM3 sample.

It was confirmed that about 100 nm of nanoparticles were evenly dispersed in both the PN sample and the PNM3 sample to which nanoparticles were added. It is judged that the constant dispersion of the nanoparticles caused their intrinsic physical properties to be exhibited on the surface of the lens, which greatly improved the optical transmittance and the tensile strength of the lens. The arithmetic mean square roughness of the lens surface decreased from 18.58 to 13.17, and the wettability of the lens increased [43]. It is believed that the wettability varies depending on the state of the surface, and the roughness of the surface also has an influence on the wettability of lens [44]. The results of the SEM and AFM analyses are shown in Figure 6 and Figure 7, respectively.

## 4. Conclusions

Neodymium oxide nanoparticles were added to the hydrogel lens to evaluate the physical properties, and the changes in the physical properties were examined according to the ratio of MA. When nanoparticles were added to the Ref sample, the tensile strength of the hydrogel lens improved, and the water content, wettability, optical transmittance, and tensile strength increased in the sample with MA further added, greatly improving the required characteristics of the hydrogel contact lens. The extractables also decreased, which increased safety of the lens. In addition, MA, a hydrophilic material, generally weakens when its water content increases, but its tensile strength improved when it was combined with the nanoparticles at a specific ratio. Therefore, it was confirmed that neodymium oxide nanoparticles and MA at an appropriate ratio were helpful in improving the functionality of the hydrogel contact lens.

## Figures and Tables

**Table 1:** Percent composition of samples (unit: wt%).

	HEMA	PEGMA	NO *	MA	EGDMA	AIBN	Total
Ref	99.30	-	-	-	0.5	0.2	100
P	90.28	9.03	-	-	0.5	0.2	100
PN	90.19	9.02	0.10	-	0.5	0.2	100
PNM1	89.29	8.93	0.10	0.98	0.5	0.2	100
PNM3	87.56	8.76	0.10	2.89	0.5	0.2	100
PNM5	85.89	8.59	0.09	4.73	0.5	0.2	100
PNM10	81.99	8.20	0.09	9.03	0.5	0.2	100

* NO: Neodymium oxide nanoparticles.

**Table 2:** pH & Extractables test of samples.

	pH Difference	Extractable Difference
Ref	0.11	2.35
P	0.08	2.64
PN	0.04	2.56
PNM3	0.07	2.00

## Data Availability

Not applicable.
